# Feature Selection Stability and Accuracy of Prediction Models for Genomic Prediction of Residual Feed Intake in Pigs Using Machine Learning

**DOI:** 10.3389/fgene.2021.611506

**Published:** 2021-02-22

**Authors:** Miriam Piles, Rob Bergsma, Daniel Gianola, Hélène Gilbert, Llibertat Tusell

**Affiliations:** ^1^Animal Breeding and Genetics Program, Institute of Agriculture and Food Research and Technology (IRTA), Barcelona, Spain; ^2^Topigs Norsvin Research Center, Beuningen, Netherlands; ^3^Department of Animal Sciences, University of Wisconsin-Madison, Madison, WI, United States; ^4^Department of Dairy Science, University of Wisconsin-Madison, Madison, WI, United States; ^5^GenPhySE, INRAE, Université de Toulouse, Castanet-Tolosan, France

**Keywords:** feature selection, stability, machine learning, genomic prediction, SNP, pigs, feed efficiency and growth

## Abstract

Feature selection (FS, i.e., selection of a subset of predictor variables) is essential in high-dimensional datasets to prevent overfitting of prediction/classification models and reduce computation time and resources. In genomics, FS allows identifying relevant markers and designing low-density SNP chips to evaluate selection candidates. In this research, several univariate and multivariate FS algorithms combined with various parametric and non-parametric learners were applied to the prediction of feed efficiency in growing pigs from high-dimensional genomic data. The objective was to find the best combination of feature selector, SNP subset size, and learner leading to accurate and stable (i.e., less sensitive to changes in the training data) prediction models. Genomic best linear unbiased prediction (GBLUP) without SNP pre-selection was the benchmark. Three types of FS methods were implemented: (i) filter methods: univariate (univ.dtree, spearcor) or multivariate (cforest, mrmr), with random selection as benchmark; (ii) embedded methods: elastic net and least absolute shrinkage and selection operator (LASSO) regression; (iii) combination of filter and embedded methods. Ridge regression, support vector machine (SVM), and gradient boosting (GB) were applied after pre-selection performed with the filter methods. Data represented 5,708 individual records of residual feed intake to be predicted from the animal’s own genotype. Accuracy (stability of results) was measured as the median (interquartile range) of the Spearman correlation between observed and predicted data in a 10-fold cross-validation. The best prediction in terms of accuracy and stability was obtained with SVM and GB using 500 or more SNPs [0.28 (0.02) and 0.27 (0.04) for SVM and GB with 1,000 SNPs, respectively]. With larger subset sizes (1,000–1,500 SNPs), the filter method had no influence on prediction quality, which was similar to that attained with a random selection. With 50–250 SNPs, the FS method had a huge impact on prediction quality: it was very poor for tree-based methods combined with any learner, but good and similar to what was obtained with larger SNP subsets when spearcor or mrmr were implemented with or without embedded methods. Those filters also led to very stable results, suggesting their potential use for designing low-density SNP chips for genome-based evaluation of feed efficiency.

## Introduction

Statistical models and methods used for predicting phenotypes or breeding values of selection candidates have an impact on the efficiency of genomic selection (GS). Machine learning (ML) methods are appealing for genomic prediction; they encompass a wide variety of techniques and models to predict outputs or to identify patterns in large datasets. Those methods do not require assumptions about the genetic determinism underlying the trait. ML is increasingly used in situations where the number of parameters is much larger than the number of observations, as it is the case for high-density genetic markers for GS. Thus, in animal and plant breeding, ML models that are non-linear in either features or parameters have been proposed to enhance genome-enabled prediction of complex traits (Gianola et al., 2006, 2011; Gianola and van Kaam, 2008).

Feature selection (i.e., selection of a subset of predictor variables, also known as features, from the input data; FS) reduces computation requirements and prevents over-fitting which occurs with high-dimensional data (Chandrashekar and Sahin, 2014). In addition, when features have a high level of redundancy, different training samples can produce different feature ranks (and therefore different models when a subset of features is selected) with the same prediction accuracy. In genetic studies, the stability of FS methods or “preferential stability” (i.e., the agreement of prediction models produced by an algorithm when trained on different training sets) is important to understand biological processes involved in the trait of interest and to design small low-cost prediction chips for GS or diagnostic (Phuong et al., 2005; Bermingham et al., 2015; Alzubi et al., 2018). Overall, it is wished to achieve a good prediction performance on independent data sets and a stable possible set of predictors, this being understood as those less sensitive to changes in the training set.

A review of FS methods can be found in Saeys et al. (2007), Chandrashekar and Sahin (2014), and Venkatesh and Anuradha (2019). All FS methods take as input a matrix of predictor variables (e.g., SNP genotypes or microarrays) for a set of samples with different output or target (i.e., the phenotype) and return a set of selected features of user-defined or tuned size. FS methods can be classified into three groups of methods: wrapper, embedded, and filter (Guyon and Elissee, 2003). Wrapper methods fit a supervised learning model using different subsets of the whole set of features, which are evaluated by a performance measurement calculated on the resulting model. Examples of wrapper methods are evolutionary FS algorithms and recursive feature elimination methods (Samb et al., 2012). Most wrapper methods are computationally infeasible for high-dimensional data sets (Saeys et al., 2007). Embedded methods perform FS as part of the model construction/fitting procedure. Some examples are least absolute shrinkage and selection operator (LASSO) regression, elastic net (Zou and Hastie, 2005), and tree-based methods (Strobl et al., 2008; Waldmann, 2016). Finally, filter methods compute a score for each feature independently of the learning algorithm and then select a set of a fixed number of them (which can be optimized) with the highest scores or those that exceed a defined threshold. Filter methods can be combined with any kind of predictive method, even methods with embedded FS. Filter methods can be univariate if they do not consider interactions between features or multivariate if they do so. As they do not rely on learning algorithms, filter methods avoid overfitting and are computationally less demanding than wrapper and embedded methods. However, using univariate methods it is possible to select redundant variables and discard features that are informative when combined with others but less informative on their own.

Measures that have been used to quantify FS stability can be classified into similarity-based and frequency-based measures (Nogueira et al., 2018). Similarity-based estimators measure stability over all pairs of feature subsets (e.g., Generalized Kalousis estimator, Kalousis et al., 2007), whereas frequency-based estimators measure stability by the frequencies of selection of each feature over the feature sets (e.g., the relative weighted consistency; Somol and Novovicova, 2010). Recently, Nogueira et al. (2018) established five desirable properties a stability estimator must have. These properties are: (i) to allow variation in the number of features selected; ii) to be a decreasing function of the variable sample variances; (iii) to be upper/lower bounded by constants not dependent on the number of features selected; (iv) to achieve its maximum only when all selected feature sets across training sets are identical and; (v) to be corrected for chance. After concluding that none of the existing stability estimators possesses all these properties, they proposed a novel one meeting these properties and provided confidence intervals and hypothesis tests on stability, which is crucial for proper comparison among FS algorithms.

Several studies from different domains have compared the predictive performance of FS methods combined with classification or prediction methods using experimental or simulated data sets. However, only few used genomic data and none evaluated the stability of the performance of the FS algorithm. For example, Gunavathi et al. (2017) and Bolón-Canedo et al. (2014) compared classification accuracy of different FS methods based on microarrays datasets. Bommert et al. (2020) compared some of the most prominent types of filter methods for FS in terms of accuracy and computing time across 16 high-dimensional classification datasets, including microarray data. The best FS methods differed among datasets, so they recommended testing several ones in each specific analysis.

The objective of our research was to explore the influence of various combinations of FS methods and learners on prediction quality and stability of models for predicting residual feed intake (RFI) from SNP genotypes, in order to find the best strategy for genetic evaluation of growing pigs at reduced genotyping cost.

## Materials and Methods

The data used was from an existing database made available by Topigs Norsvin (Beuningen, Netherlands). No Animal Care Committee approval was necessary for our purposes.

### Animals

Animals were 5,708 boars from a terminal sire line originated from 217 boars and 1,120 sows from Topigs Norsvin (Beuningen, Netherlands). All animals were born and raised in two Specific Pathogen Free nucleus farms, located in Netherlands and France, with semen exchange between farms being frequent.

#### Phenotypes

Nucleus farms were equipped with IVOG feeding stations (INSENTEC, Marknesse, Netherlands) that register individual feed intake of group housed pigs. All pigs had ear tags with unique numbering; individual feed intake records were available for all pigs for each day on the test. The pigs had *ad libitum* access to water and to a commercially available diet until the end of the performance test.

Average daily gain (ADG) was measured between the beginning (median age of 68days and median weight of 31Kg) and end of the test (median age of 155days and median weight of 130Kg). Only records from boars starting the test period between 50 and 105days of age and remaining on the test between 60 and 120days were retained.

Backfat thickness (BFT) was determined ultrasonically on live animals (US-fat in mm) at the end of the test period. Metabolic weight (MW; g) was calculated as: $MW={\left(\frac{W_{start}+W_{end}}{2}\right)}^{0.75}$, where $W_{start}$ and $W_{end}$ are the weights at the beginning and end of the test period, respectively.

Multivariate outlier records of ADG, daily feed intake (DFI), BFT, and MW were identified and removed within batch and farm when the squared Mahalanobis distance away from the center of the distribution was >12 (Drumond et al., 2019). Then, RFI was estimated as the residual of a phenotypic linear regression of DFI on ADG, BFT, and MW. Thus, for animal *i*th:

$${\mathrm{DFI}}_{i}={\text{β}}_{1}\times {\mathrm{ADG}}_{i}+{\text{β}}_{2}\times {\mathrm{BFT}}_{i}+{\text{β}}_{3}\times {\mathrm{MBW}}_{i}+{\mathrm{RFI}}_{i}$$

Subsequently, RFI records were pre-adjusted by macro-environmental effects fitting a linear model which included the fixed effects of age at the start of the test (Age, covariate), duration of the performance test (Length, covariate), and the combination of farm and batch (FarmBatch, 46 levels). The FarmBatch effect resulted from the combination of two farms and 2-month period batches. All FarmBatch levels retained for the analyses had at least 10 records. Thus, for animal *i*th and level of FarmBatch *j*th:

$${\mathrm{RFI}}_{\mathrm{ij}}={\mathrm{FarmBatch}}_{j}+{\text{β}}_{1}\times {\mathrm{Age}}_{i}+{\text{β}}_{2}\times {\mathrm{Length}}_{i}+e_{\mathrm{ij}}$$

The adjusted RFI records were obtained after subtracting the estimates of these systematic environmental effects from the values of the original trait. From here onwards, we refer to adjusted RFI (i.e., *e_ij_*) as RFI. Both linear models were fitted using the using lm() function (R Development Core Team, 2020).

#### Genotypes

Animals were genotyped using the Illumina Porcine SNP60 BeadChip (Illumina Inc., San Diego). Assuming an additive allele substitution effect, genotypes were arbitrarily coded to 0, 1, and 2 for the homozygote for the minor allele, heterozygote, and other homozygote, respectively. SNPs with a call rate lower than 0.90 and a minor allele frequency lower than 0.05 were removed. Boars with a call rate lower than 0.90 and parent-offspring pairs that displayed Mendelian inconsistencies were discarded. After this quality control, 46,610 SNPs were retained to pursue the analyses. Zero and near-zero-variance SNPs were identified and removed with the “nearZeroVar” function which removes predictors that have a unique value or have very few unique values relative to the number of samples, and the ratio of the frequency of the most common value to the frequency of the second most common value is 95/5 (Caret R package, Kuhn, 2008). Subsequently, the “findCorrelation” function (Caret R package, Kuhn, 2008) with a cut-off = 0.8 was used to diminish high pair-wise correlations between features. After this genotype edition, 9,523 SNPs were retained.

#### Model Fitting and Prediction

**Figure 1:**
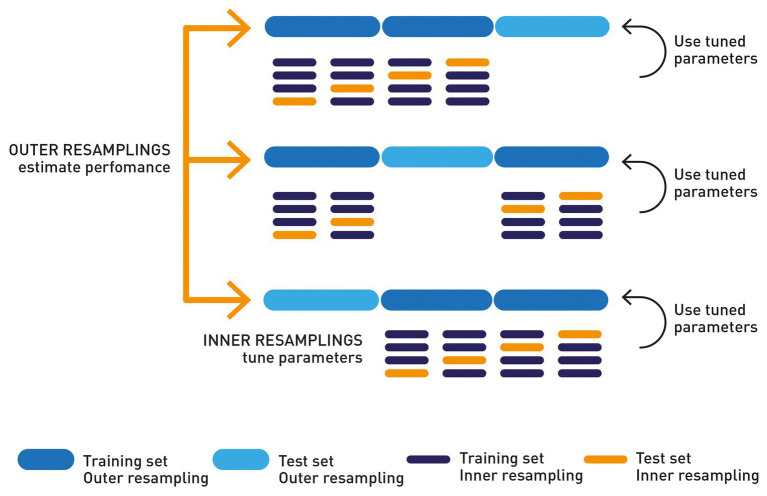
Nested resampling diagram.

Models were fitted using individual genotypes as predictor variables and individual RFI records as output or target variable. For each combination of prediction method (i.e., learner) and SNP subset size, model fitting and (hyper)parameter optimization were conducted with a nested cross-validation (Figure 1). Nested cross-validation allows estimating the generalization error of the underlying model and its (hyper)parameter search (Bischl et al., 2016). It consists of several training-validation and testing dataset splits. An outer 10-fold cross-validation using all data was performed using nine equal-size parts of the original data sets for training the model, and the remaining one for testing. Within each outer training set, features (i.e., SNPs) were standardized and FS was performed using several methods and for a varying number of selected features (50, 250, 500, 750, 1,000, and 1,500). Also, within each outer training set, an inner six-fold cross-validation was implemented for tuning the hyper-parameters of the model. Hyper-parameter values were chosen based on a mean square error on the validation set of this inner cross-validation. The model was finally fitted to the whole training set using the optimal hyper-parameters. Same data split (i.e., same data subsets) was used across combinations of learners and datasets to compare prediction performance in the same conditions regarding data structure and composition.

In what follows, a description of the FS methods and learners implemented is provided first. Then, measurements of quality and stability of the predictions and of the selected features are defined.

#### Feature Selection Methods

Several filter and embedded FS methods (Saeys et al., 2007), and combinations hereof were used to rank and select the most relevant SNPs for predicting the target trait.

##### Filter Methods

The filter methods implemented were either univariate or multivariate to account for interactions between features. Consider a dataset of *N* records of *p* features (i.e., predictor variables, SNPs in this study which are considered to be continuous variables) in a set *S* of features and a target variable of interest *Y*. Five filter methods were implemented to rank the available SNPs according to their relevance for prediction of the target trait (Bommert et al., 2020): (i) Sort features with the Spearman’s rank correlation (spearcor) between each feature *X* and *Y*. (ii) Univariate decision tree (univ.dtree) resamples a decision tree for each feature individually. The resampling performance is used as a filter score to rank features. (iii) Maximal relevance minimal redundancy filter (mrmr) is based on the concept of mutual information of two variables, defined as $I\left(\mathrm{Y;X}\right)=H\left(Y\right)-H\left(\mathrm{Y|X}\right)$ where $H\left(Y\right)=-\int f\left(Y\right)\log f\left(Y\right)\mathrm{dy}$ is the differential entropy and $H\left(\mathrm{Y|X}\right)=-\int \int f\left(\mathrm{Y,X}\;\right)\mathrm{logf}\left(\mathrm{Y|X}\;\right)\mathrm{dy}\;\mathrm{dx}\;$ is the conditional differential entropy (Ding and Peng, 2005). The entropy measures the uncertainty of the variable. The mutual information of two variables can be interpreted as the decrease in uncertainty about *Y* conditional on knowing *X* or as the amount of information shared by both variables since $I\left(\mathrm{Y;X}\right)=I\left(\mathrm{X;Y}\right)$. Filter mrmr uses the score $I\left({\mathrm{Y;X}}_{k}\right)-\frac{1}{\left|S\right|}\underset{X_{j}\in S}{\sum }I\left(X_{k}{\mathrm{;X}}_{j}\right)$ where term $I\left({\mathrm{Y;X}}_{k}\right)$ measures the relevance of the *k*th feature *via* the information this feature has about *Y*, while the term $\frac{1}{\left|S\right|}\underset{X_{j}\in S}{\sum }I\left(X_{k}{\mathrm{;X}}_{j}\right)$ measures its redundancy by the mean information that the feature shares with other *j*th features in the set *S* of size $\left|S\right|$. Therefore, the variables with the highest values of the score are those that have the maximum relevance and minimum redundancy with the other variables. (iv) Conditional permutation importance for correlated predictors (Strobl et al., 2008) of fitted random forest (cforest) uses a randomly permuted feature *X_k_* to predict the response and to evaluate the difference in prediction accuracy before and after permuting that predictor *X_k_*. If the original *X_k_* variable is associated with the response, this permutation will lead to a decrease in prediction accuracy. Its advantage over univariate screening methods is that it covers the impact of each predictor variable individually, as well as in multivariate interactions with the other predictor variables. Conditional permutation importance (Strobl et al., 2008) was chosen to rank the markers because of the existence of correlation patterns among them. In this method, the permutation is performed within groups of observations that are defined by the values of the remaining predictor variables. (v) Random selection of SNPs (random), used as benchmark.

##### Embedded Methods

In the embedded methods the search for an optimal subset of features is done within the prediction model. Like wrapper methods, they are specific to a learning algorithm but less computationally demanding. Embedded methods used here were LASSO regression (LR, Park and Casella, 2008) and elastic net (ENET, Zou and Hastie, 2005), as explained in the section below.

#### Learners

Ridge regression (RR), LR, ENET, support vector machine for regression (SVM), and gradient boosting (GB) were used for predicting RFI records. Genomic best linear unbiased prediction (GBLUP, VanRaden, 2008) was used as benchmark.

Elastic net (Zou and Hastie, 2005) is originally a regression method that combines *L1* ($\lambda_{1}\times \left[\sum_{j=1}^{p}{\text{β}}_{j}^{2}\right]$) and *L2* ($\lambda_{2}\times \left[\sum_{j=1}^{p}\left|{\text{β}}_{j}\right|\right]$) penalties of ridge and LASSO in a mixture of the two. Parameters $\lambda_{1}$ and $\lambda_{2}\;$ control the strength of the *L1* and *L2* penalties and ${\text{β}}_{j}$ is the regression coefficient on SNP *j*th. The ENET penalty is: $\lambda \times \left(\left(1-\alpha \right)\times \left[\sum_{j=1}^{p}\left|{\text{β}}_{j}\right|\right]+\alpha \times \left[\sum_{j=1}^{p}{\text{β}}_{j}^{2}\right]\right)$, where $\alpha =(\frac{\lambda_{2}}{\lambda_{1}+\lambda_{2}}$).Thus,

$\hat{\text{β}}=\arg min_{\text{β}}\left({\left\|y-X\text{β}\right\|}^{2}\right)+\lambda \times \left(\left(1-\alpha \right)\times \left[\sum_{j=1}^{p}\left|{\text{β}}_{j}\right|\right]+\alpha \times \left[\sum_{j=1}^{p}{\text{β}}_{j}^{2}\right]\right)$, where *y* is the vector of adjusted phenotypes of dimension N×1, *X* is the matrix of standardized genotypes of dimension N×p and $\text{β}=\left\{{\text{β}}_{j}\right\}$ is the vector of regression coefficients of dimension p×1. The genotypes were standardized to have a mean of 0 and a standard deviation of 1.

Elastic net is an embedded method of FS because it allows selecting a subset of predictor variables out of *p* candidates. When N≪p, ENET can select more than *N* predictor variables. This learner was implemented with the various SNP subset sizes, and with the full SNP set as well (9,523 SNPs). The function “cv.glmnet” from the “glmnet” R package (Friedman et al., 2010) was used to fit ENET. The value of *α* was tuned by testing values from 0 (i.e., a LR model) to 1 (i.e., a RR model) in increments of 0.1; the optimal *λ* parameter was found by cross-validation. Variable importance in the different fitted models was measured as the regression coefficients of each standardized predictor variable.

Support vector machine aims at identifying a function that has a maximum deviation *ε* from the observed values (*Y*) and has a maximum margin for the set of prediction variables (*S*). A review of this method can be found in Smola and Schölkopf (2004). The power of the SVM resides in a particular component known as kernel. One of the most used kernel is the Gaussian Radial Basis Function (RBF) because almost every surface can be obtained with it (Christianini and Shawe-Taylor, 2000). One of the main parameters in a SVM is the “cost parameter” (*C*), which is a trade-off between the prediction error and the simplicity of the model. The other hyper-parameter (*γ*) of SVM enters into the Gaussian function inside the RBF kernel. Performance of SVM is very sensitive to changes in *γ* parameter. Tested values for *C* were 0.001, 0.1, 1, 5, and 10, and for *γ* 0.005, 0.05, 0.5, and 5. The “e1071” R package was used for the analyses (Meyer et al., 2019).

Gradient boosting or GB (Mason et al., 1999) is an ensemble method because it uses several fast and easy computation learning algorithms to get a better predictive performance than the one that could be obtained by using the algorithms individually. Predictors are combined sequentially by applying some shrinkage to each. Details on GB can be found in Hastie et al. (2009). For implementing GB, three hyper-parameters were tuned: depth of tree (10, 12, 15, 17), a learning rate that controls the size of the steps in the gradient descent process (0.01 and 0.02), and the number of trees (500, 1,000, and 3,000 trees). The “gbm” R package was used (Greenwell et al., 2019).

A Bayesian GBLUP was used as a reference employing the same outer training and testing datasets partitions as those used with the other learners. In Bayesian GBLUP, the model was y=1μ+u+ε the vector of genomic breeding values, $u=\left\{u_{i}\right\}$, are assumed to be normally distributed as $p\left(u|\sigma_{u}^{2}\right)\sim N\left(0,G\sigma_{u}^{2}\right)$. The $\sigma_{u}^{2}$ is the additive genomic variance and the genomic relationship matrix (*G*) was computed from the 46,610 SNPs as $G=\frac{\left(M-E\right)\left(M-E\right)\prime }{2\sum_{j=1}^{p}q_{j}\left(1-q_{j}\right)}$ (VanRaden, 2008). Marker genotypes in M were previously centered by subtracting the average allele frequencies at each locus (i.e., $2q_{j}$ from each element on column j of E where qj is the allelic frequency of the major allele of the jth SNP. Since allele frequencies at each locus from the base population were not available, they were computed directly from the available data. The distribution for random residuals was assumed to be $p\left(\varepsilon |\sigma_{\varepsilon }^{2}\right)\sim N\left(0,Ι\sigma_{\varepsilon }^{2}\right)$. Priors assumed for $\sigma_{u}^{2}$ and $\sigma_{\varepsilon }^{2}$ were scaled inverse $-\chi^{2}$ distributions with degrees of freedom $d_{l}$ and scale factor *S_l_*: $p\left(\sigma_{l}^{2}{\mathrm{|d}}_{l}{\mathrm{,S}}_{l}\right)\sim \chi^{-2}\left(\sigma_{l}^{2}{\mathrm{|d}}_{l}{\mathrm{,S}}_{l}\right)$ for $1\varepsilon \left\{\mathrm{u,}\varepsilon \right\}$.

The Gibbs2f90 software (Misztal, 1999) was used to implement this method. Flat priors were assumed for $\sigma_{u}^{2}$ and the residual variance. Single chains of 250,000 iterations were run by discarding the first 25,000. The number of discarded samples was, in all folds, larger than the required burn-in that was determined by visual inspection of the chains. Samples of parameters of interest were saved every 10 iterations and their posterior means were retained for each training/testing partition of the dataset for later comparison. Effective sample size was larger than 700 for all the parameters of the model.

#### Quality of Prediction and Stability of Feature Selectors

The objective was to find the best combination of FS method and learner to obtain the smallest and most stable SNP subset that leads to the most accurate prediction.

The quality of trait prediction was evaluated for accuracy, as the median of the Spearman correlation (SC) between observed (i.e., adjusted phenotypes) and predicted trait across the 10 outer testing sets, and for stability/generality of results, as the interquartile range (IQR) of those values.

Stability of FS algorithms measures how variation in the training sample produces a change in the selected feature subset (Kalousis et al., 2007). If FS is performed setting a threshold for a number of the most important features for prediction based on a weight, score, or rank assigned to each feature, preferential stability can be measured as the mean Pearson’s correlation (PC) or as the SC between all pairs of weighting-scoring and ranking values, respectively, obtained in different training sets. When FS is performed by a procedure that does not involve any weight or rank (i.e., using embedded and wrapper methods), preferential stability can be measured as the amount of overlap between two sets of an arbitrary size (Generalized Kalousis, Kalousis et al., 2007). All those measures require the equal size of the feature subsets. To compare the stability of subsets of varying sizes, as obtained with embedded methods, Somol and Novovicova (2010) introduced the relative weighted consistency measure. Relative Weighted Consistency and Generalized Kalousis do not meet the properties (iv) and (v), respectively, for a proper stability estimator established by Nogueira et al. (2018) who proposed a new stability estimator $\hat{\Phi }\left(S\right)$ (NOG) defined as:

$$\hat{\Phi }\left(S\right)=1-\frac{\frac{1}{p}\sum_{i=1}^{p}\sigma_{f_{i}}^{2}}{E\left[\frac{1}{p}\sum_{i=1}^{p}\sigma_{f_{i}}^{2}\left|\;\right)H_{0}\right]}=1-\frac{\frac{1}{p}\sum_{i=1}^{p}\sigma_{f_{i}}^{2}}{\frac{\bar{d}}{p}\left(1-\frac{\bar{d}}{p}\right)}$$

For $T=\left\{f_{1}{\mathrm{,f}}_{2},\ldots {\mathrm{,f}}_{p}\;\right\}$ being the whole set of features of size *p* (i.e., 9,523 in our study) and $S=\left\{S_{1}{\mathrm{,S}}_{2},\ldots {\mathrm{,S}}_{n}\;\right\}$ being a system of *n* > 1 subsets $S_{j}=\left\{f_{i}\mathrm{|i}=1,\;\ldots {\mathrm{,d}}_{j}{\mathrm{, f}}_{i}\in {\mathrm{Y, d}}_{j}\in \left\{1,2,\ldots \mathrm{,p}\right\}\;\right\}$ with *j* = 1, 2,…, *n* obtained from *n* runs of the FS algorithm (*n* = 10 in our study), $\sigma_{f_{i}}^{2}=\frac{n}{n-1}{\hat{p}}_{f_{i}}\left(1-{\hat{p}}_{f_{i}}\right)$ is the unbiased sample variance of the *i*th SNP,$\;F_{f_{i}}$ is the frequency of the feature *f_i_*, ${\hat{p}}_{f_{i}}=\frac{1}{n}F_{f_{i}}$ is the mean of the former, and $\bar{d}$ is the average number of features selected over the *n* feature sets. To correct for similarity between feature sets due to chance, the estimator is rescaled by its expected value under the Null model of random FS ($H_{0}$). NOG ranges from 0 to 1. Confidence intervals for the population stability $\Phi \left(S\right)$ were approximated with a 0.05 significance level. Refer to Nogueira et al. (2018) for further details. In this study, PC, SC, and NOG were used as stability measurements.

## Results

Results (Singleton, 2001) refer to prediction of yet-to-be observed individual RFI based on pigs’ genotypes. Prediction performances correspond to the 10 best configurations obtained in the 10-outer cross-validation folds. Notice that FS was done by cross-validation in each of the 10-outer training folds. Therefore, for each FS method and learner, there are 10 subsets of selected features and 10 prediction performances obtained with those subsets, allowing a measurement of the stability of the FS method, as well as a measurement of the dispersion of prediction accuracy.

### Prediction Quality: Accuracy and Stability

**Figure 2:**
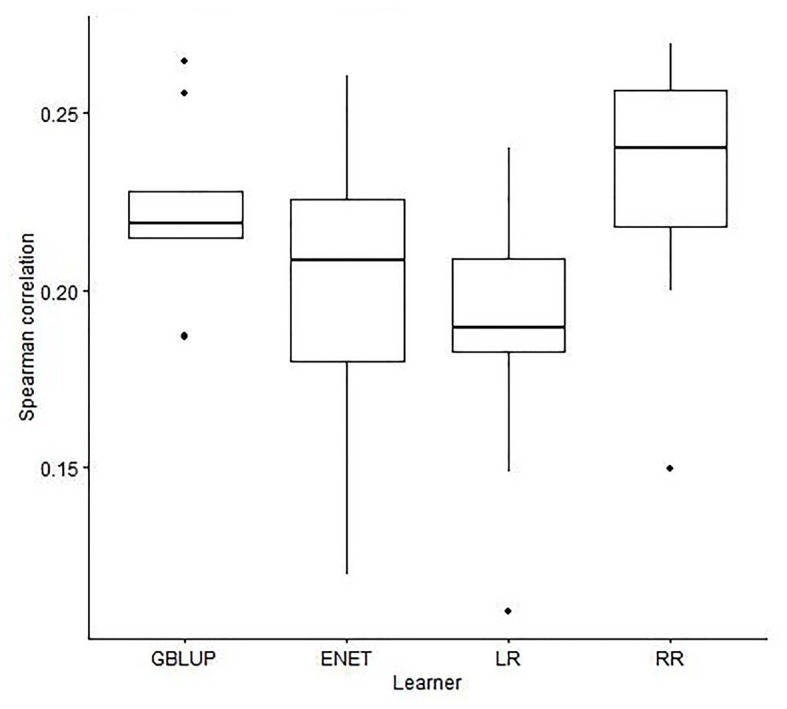
Boxplots for the Spearman correlation obtained in 10-outer testing sets with a genomic best linear unbiased predictor (GBLUP), elastic net (ENET), least absolute shrinkage and selection operator regression (LR), and ridge regression (RR) with no feature selection method used to select SNPs.

**Figure 3:**
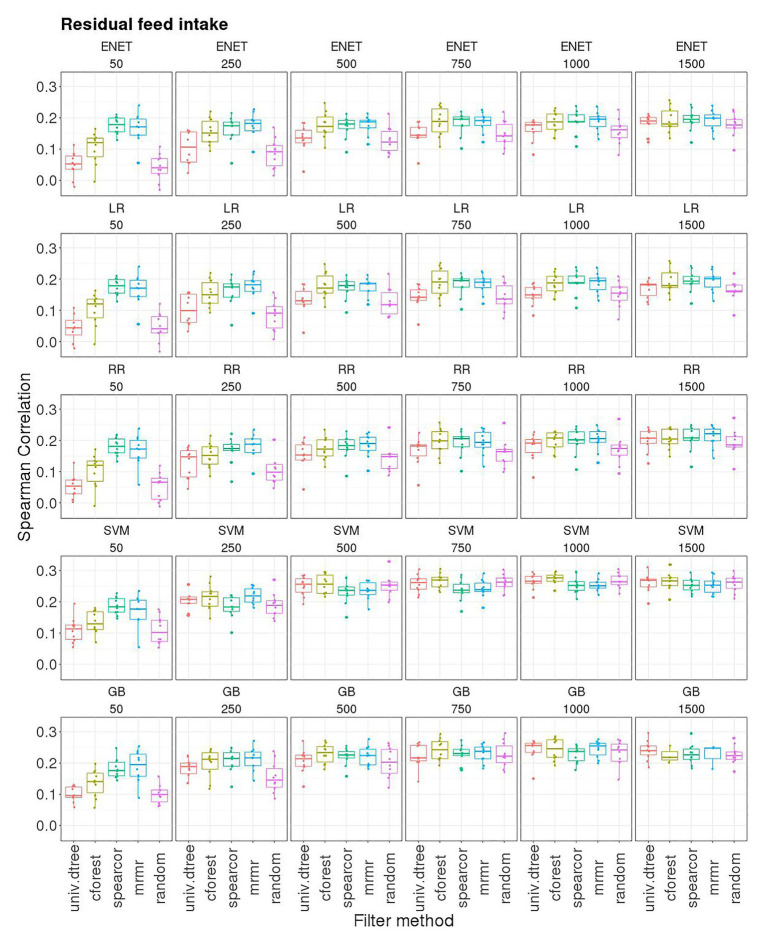
Boxplots for the Spearman correlation obtained in 10-outer testing sets with support vector machine for regression (SVM), gradient boosting (GB), elastic net (ENET), least absolute shrinkage and selection operator regression (LR), and ridge regression (RR) with 50, 250, 500, 750, 1,000, and 1,500 SNP subsets selected with different filter methods. Filter methods: Maximum relevance minimum redundancy (mrmr), random forest (cforest), Spearman’s correlation (spearcor), univariate decision tree (univ.dtree), and random selection (random).

Figures 2, 3 show boxplots for SC obtained in the 10-outer testing sets with GBLUP, ENET, LR, and RR without pre-selection of SNPs (Figure 2), and with SVM, GB, ENET, LR, and RR with pre-selection of SNPs performed using the various filter methods (Figure 3). No results were obtained with SVM and GB without pre-selection of SNPs due to numerical problems.

When the prediction was performed with GBLUP the median (IQR) of SC was 0.22 (0.01; Figure 2), not significantly different from the obtained with RR (median of SC = 0.22) and ENET (median of SC = 0.19) with 9,523 SNPs. However, it was more stable for GBLUP since IQR was 0.04 and 0.03 for RR and ENET, respectively (Figure 2). LASSO regression had the poorest performance in terms of accuracy and stability of results [median of SC = 0.19 (0.03)].

Elastic net, RR, and LR combined with different filter methods had the same pattern of prediction performance (Figure 3). For subset sizes smaller than 500, prediction accuracy was smaller than with GBLUP when univ.dtree and random selection of SNPs were used as filter methods. However, the same accuracy as GBLUP was obtained when spearcor and mrmr filters were used, even with only 50 SNPs, while cforest filter led to an intermediate predictive performance. The effect of the filter on SC decreased with an increasing number of SNPs up to 1,500, for which SC was not statistically different across filters. Random selection of SNPs led to very poor performances with subset sizes of 50 and 250 SNPs. When more than 250 SNP were used as predictor variables, with SVM and GB used as learner SC of the random filter was the same as the one attained with other filter methods, whereas it remained lower than the other filters when ENET, RR, and LR were used.

Globally, SC attained with SVM and GB increased as the number of SNPs increased up to 500 SNPs, and then remained at about the same level. The stability of results measured as IQR of SC followed the same pattern. However, when spearcor or mrmr were used to perform pre-selection of SNPs, the SC attained with 50 SNPs was close to that attained with 500 or more SNPs: 0.18 (0.04) and 0.18 (0.06) with spearcor and mrmr combined with SVM, respectively; 0.18 (0.04) and 0.20 (0.07) with spearcor and mrmr combined with GB, respectively. The highest median SC was obtained with SVM [0.28 (0.02)] and GB [0.27 (0.04)] using a subset with the 1,000 best-ranked SNPs according to cforest (Figure 3). Performance obtained with SVM and GB with just 750 SNPs was in all cases equal or superior to the median SC obtained using GBLUP (0.22), although results were slightly more stable with GBLUP (IQR: 0.02 for the best model with SVM or GB compared to 0.01 for GBLUP).

### Feature Selection Stability

**Figure 4:**
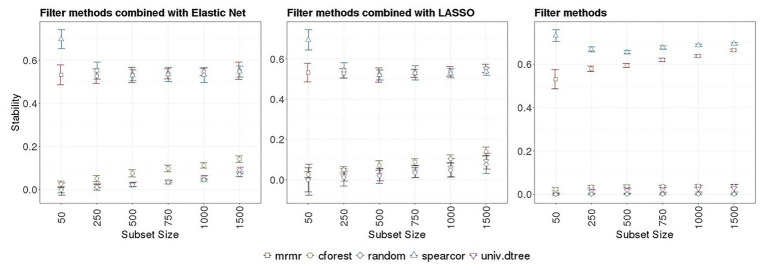
Left and middle panels: Boxplots for Nogueira et al. (2018) stability estimator obtained from embedded methods implemented with different SNP subsets sizes and several filter methods in 10 outer-training folds. Right panel: Boxplots for Nogueira et al. (2018) stability estimator of different SNP subset sizes obtained with different filter methods in the 10-outer training folds. Subsets sizes: 50, 250, 500, 750, 1,000, and 1,500 SNPs. Filter methods: Maximum relevance minimum redundancy (mrmr), random forest (cforest), Spearman’s correlation (spearcor), univariate decision tree (univ.dtree), and random selection (random).

Stability of FS methods for prediction of RFI with ENET and LR implemented with SNP subsets obtained with or without pre-selection with various filter methods are presented in Tables 1 and 2, respectively. Boxplots for NOG values from ENET and LR by filter method in the 10 subsets are shown in the left and middle panels of Figure 4, whereas boxplots for NOG values from just filter methods are in the right panel of Figure 3. According to the scale defined by Nogueira et al. (2018), without pre-selection of SNPs the stability estimator for ENET and LR was intermediate to good (0.57 and 0.56 for ENET and LR, respectively; Tables 1 and 2). In this case, the median (IQR) number of selected SNPs for ENET was 717 (11) out of 9,523 SNPs (Table 1), while LR performed a stronger but less stable selection, retaining only 269 (111) SNPs (Table 2). With pre-selection of the most important features, ENET and LR removed a considerable part of them and showed differences in FS stability among filter methods and subset sizes (Tables 1 and 2, Figure 4). As expected, the number of SNPs selected in regularized regression (ENET and LR) was smaller for random SNP pre-selection than for other filters, ranging from 42% for subset size of 50 to 2.2% for subset sizes of 1,500. However, it ranged from 100% for subset sizes of 50 to 40% for subset sizes of 1,500 SNPs when pre-selection was performed with mrmr. Univariate decision tree performed like random selection whereas in cforest and spearcor the selected percentage was between random and mrmr filters. Elastic net and LR produced similar patterns of FS stability with increasing subset size. The most stable subsets were obtained with spearcor, with excellent values when the subset size of pre-selected SNPs was 50 (0.70 and 0.69 for ENET and LR, respectively, Figure 4, Tables 1 and 2). For other subset sizes, no differences in stability were found between spearcor and mrmr, or across subset sizes within filter, with NOG values ranging from 0.52 to 0.55. All other filter methods (i.e., cforest, univ.dtree, or random) gave very poor FS stabilities. Univariate decision tree for SNP pre-selection combined with ENET and LR had null stabilities, with magnitudes that were similar to a random selection. Cforest combined with either ENET or LR slightly improved FS stability up to 0.14 with 1,500 SNPs, which was significantly different from random selection, but very unstable FS and far from the most stable methods.

**Table 1:** Stability of feature selection methods for prediction of residual feed intake with elastic net.

Subset size^1^	Filter^2^	NSNPs^3^	PDF^4^	medianSel^5^	IQRSel^6^	NOG^7^
50	mrmr	499	0.30	50	0	0.53
	cforest	458	0.89	47	3	0.03
	spearcor	445	0.20	45	2	0.70
	univ.dtree	219	0.98	22	6	0.00
	random	209	1.00	21	4	0.00
250	mrmr	2,163	0.28	217	8	0.53
	cforest	1,641	0.79	167	12	0.05
	spearcor	1,541	0.28	155	8	0.55
	univ.dtree	900	0.89	93	8	0.02
	random	854	0.91	87	15	0.01
500	mrmr	3,294	0.28	330	20	0.53
	cforest	2,175	0.71	223	35	0.08
	spearcor	2,597	0.27	263	11	0.53
	univ.dtree	1,501	0.84	154	14	0.02
	random	1,547	0.85	148	19	0.02
750	mrmr	4,258	0.27	431	19	0.54
	cforest	2,493	0.65	250	16	0.10
	spearcor	3,426	0.27	344	21	0.53
	univ.dtree	2,105	0.79	213	13	0.04
	random	2,039	0.80	202	18	0.03
1,000	mrmr	5,059	0.26	511	13	0.55
	cforest	2,879	0.62	292	18	0.11
	spearcor	4,074	0.27	412	20	0.53
	univ.dtree	2,546	0.73	253	11	0.06
	random	2,455	0.75	244	7	0.05
1,500	mrmr	5,929	0.26	593	61	0.55
	cforest	3,557	0.56	355	7	0.14
	spearcor	4,973	0.27	497	23	0.55
	univ.dtree	3,241	0.64	326	16	0.09
	random	3,217	0.66	324	18	0.07
9,523	none	7,193	0.25	717	11	0.57

1 Subset size = number of selected features.

2 Filter method = Maximum relevance minimum redundancy (mrmr); Random forest (cforest); Spearman’s correlation (spearcor); Univariate decision tree (univ.dtree); Random selection (random).

3 NSNPs = Total number of SNPs pre-selected in the 10 subsets.

4 PDF = Proportion of distinct features in the 10 subsets.

5 medianSel =Mean number of selected SNPS in the 10 subsets.

6 IQRSel = Interquartile range of the number of selected SNPS in the 10 subsets.

7 NOG = Nogueira et al. (2018) stability estimator.

**Table 2:** Stability of feature selection methods for prediction of residual feed intake with LASSO regression.

Subset size^1^	Filter^2^	NSNPs^3^	PDF^4^	medianSel^5^	IQRSel^6^	NOG^7^
50	mrmr	499	0.30	50	0	0.53
	cforest	454	0.89	46	5	0.03
	spearcor	441	0.20	44	3	0.69
	univ.dtree	151	0.99	24	10	0.00
	random	189	0.99	23	12	0.00
250	mrmr	2,096	0.28	209	8	0.53
	cforest	1,693	0.79	172	13	0.05
	spearcor	1,575	0.28	158	12	0.54
	univ.dtree	804	0.90	83	18	0.02
	random	740	0.92	80	48	0.01
500	mrmr	3,420	0.29	347	23	0.52
	cforest	2,287	0.72	239	39	0.07
	spearcor	2,560	0.28	260	9	0.52
	univ.dtree	1,292	0.86	141	40	0.02
	random	1,332	0.86	151	63	0.02
750	mrmr	4,333	0.28	437	24	0.53
	cforest	2,646	0.67	275	35	0.09
	spearcor	3,339	0.27	338	21	0.53
	univ.dtree	1,667	0.79	165	40	0.04
	random	1,745	0.80	160	57	0.03
1,000	mrmr	5,125	0.27	515	23	0.54
	cforest	3,008	0.63	321	53	0.10
	spearcor	3,978	0.27	394	15	0.53
	univ.dtree	1,750	0.74	193	21	0.06
	random	1,879	0.76	192	62	0.05
1,500	mrmr	5,978	0.26	595	21	0.55
	cforest	3,478	0.56	341	8	0.14
	spearcor	5,096	0.27	506	12	0.54
	univ.dtree	2,270	0.66	235	19	0.09
	random	2,200	0.68	235	80	0.08
9,523	none	2,963	0.27	269	111	0.56

1 Subset size = number of selected features.

2 Filter method = Maximum relevance minimum redundancy (mrmr); Random forest (cforest); Spearman’s correlation (spearcor); Univariate decision tree (univ.dtree); Random selection (random).

3 NSNPs = Total number of SNPs pre-selected in the 10 subsets.

4 PDF = Proportion of distinct features in the 10 subsets.

5 medianSel = Mean number of selected SNPS in the 10 subsets.

6 IQRSel = Interquartile range of the number of selected SNPS in the 10 subsets.

7 NOG = Nogueira et al. (2018) stability estimator.

Stability measurements were slightly smaller when filter methods were combined with embedded methods than when only filter methods were implemented, for all subset sizes (Tables 1 and 2 vs. Table 3). According to the scale defined by Nogueira et al. (2018), when only filter methods were used, the best FS stability was obtained with spearcor for any subset size ranging from 0.73 to 0.69 when 50 and 1,000–1,500 SNPs were pre-selected, respectively (Figure 4, right panel). Maximum relevance minimum redundancy also had a good FS stability. Unlike for spearcor, FS stability of mrmr increased with an increasing number of SNPs, from 0.53 to 0.66 with 50 and 1,500 SNPs, respectively. Univariate decision tree and cforest showed null stability, as random selection, with a maximum of 0.04 for cforest with 1,500 SNPs, and marginal improvement with an increase of subset sizes.

**Table 3:** Stability of filter methods for prediction of residual feed intake.

Subset size^1^	Filter method^2^	NSNPs^3^	PDF^4^	NOG^5^
50	mrmr	500	0.30	0.53
	cforest	500	0.90	0.02
	spearcor	500	0.19	0.73
	univ.dtree	500	0.96	0.00
	random	500	0.98	0.00
250	mrmr	2,500	0.26	0.58
	cforest	2,500	0.80	0.04
	spearcor	2,500	0.23	0.67
	univ.dtree	2,500	0.85	0.01
	random	2,500	0.88	0.00
500	mrmr	5,000	0.25	0.59
	cforest	5,000	0.72	0.04
	spearcor	5,000	0.21	0.66
	univ.dtree	5,000	0.75	0.02
	random	5,000	0.79	0.00
750	mrmr	7,500	0.22	0.62
	cforest	7,500	0.65	0.04
	spearcor	7,500	0.20	0.68
	univ.dtree	7,500	0.67	0.02
	random	7,500	0.71	0.00
1,000	mrmr	10,000	0.21	0.64
	cforest	10,000	0.59	0.04
	spearcor	10,000	0.19	0.69
	univ.dtree	10,000	0.60	0.03
	random	10,000	0.64	0.00
1,500	mrmr	15,000	0.19	0.66
	cforest	15,000	0.49	0.04
	spearcor	15,000	0.18	0.69
	univ.dtree	15,000	0.50	0.03
	random	15,000	0.52	0.00

1 Subset size = number of selected features.

2 Filter method = Maximum relevance minimum redundancy (mrmr); Random forest (cforest); Spearman’s correlation (spearcor); Univariate decision tree (univ.dtree); Random selection (random).

3 NSNPs = Total number of SNPs selected in the 10 subsets.

4 PDF = Proportion of distinct features in the 10 subsets.

5 NOG = Nogueira et al. (2018) stability estimator.

Table 4 shows the mean of the PC and SCs over all pairs of feature scores in the 10 outer training sets obtained with the different filter methods for FS. The highest PC and SCs between the scores obtained for each SNP across training sets were obtained with mrmr and spearcor, which indicates the highest stability in the selection of relevant features for prediction across outer training sets. Tree-based methods (univariate or multivariate) exhibited low stability in the selection of predictor variables.

**Table 4:** Mean of the Pearson and Spearman correlation over all pairs of feature scores in the 10-outer training sets obtained with different filter methods for feature selection for prediction of residual feed intake.

Filter method^1^	Pearson	Spearman
mrmr	0.875	0.893
cforest	0.025	0.015
spearcor	0.863	0.802
univ.dtree	0.062	0.059
random	0.001	0.001

1 Filter method = Maximum relevance minimum redundancy (mrmr); Random forest (cforest); Spearman’s correlation (spearcor); Univariate decision tree (univ.dtree); Random selection (random).

The same analyses were performed for ADG with similar results. The corresponding tables and figures showing the results obtained for this trait are provided in Supplementary Material.

## Discussion

It is well known that classification or prediction with high dimensional data is computationally demanding and may produce overfitted models with poor prediction quality that are difficult to interpret (Huang, 2014). Therefore, the search of effective FS algorithms is important, and it is an active area of research, despite overfitting can be avoided *via* regularization in some models. Such algorithms are required to develop prediction models that are accurate and insensitive to small changes in the training data. Many authors have addressed the question of the sensitivity of FS methods with respect to small changes in the training data in different domains of research. Kalousis et al. (2007) were the first to consider the stability of FS procedures. Their research was followed by several publications in application areas where stability is critical, such as microarray classification or molecular profiling and by studies addressing how to quantify stability (Davis et al., 2006; Kuncheva, 2007; Jurman et al., 2008; Zucknick et al., 2008; Zhang et al., 2009; Somol and Novovicova, 2010; Goh and Wong, 2016). The measurement of stability is important because it indicates how much the output of an algorithm can be trusted by capturing the underlying mechanism. This is very important in many biological and biomedical domains, and genetics applied to livestock production is not an exception. Here, an objective could be, for example, to design low-density SNP chips for GS, or to assign further resources to the search of genes with a major effect on important production traits.

In this research, several univariate and multivariate algorithms combined with parametric and non-parametric learners were applied to the prediction of RFI of growing pigs from high-dimensional genomic data (60K SNP chip). The objective was to find the best combination of feature selector, subset size, and learner leading to as high as possible accurate and stable predictions. GBLUP with no SNP selection beyond the standard quality control was the benchmark. Three types of FS methods were implemented: (i) filter methods: univariate (univ.dtree, spearcor) or multivariate (cforest, mrmr) and a random selection filter as benchmark; (ii) embedded methods: ENET and LR; (iii) the combination of filter and embedded methods. Regularized regression using RR, which does not perform FS, was also used as an intermediate option between no and strong FS. In addition, SVM and GB, considered to be among the most efficient ML methods, were implemented, but only with the SNP pre-selection performed with filter methods. These two methods have been successfully used in various fields (James et al., 2013; Attewell et al., 2015) including livestock and plant breeding (Moser et al., 2009; Long et al., 2011; González-Recio et al., 2014; Montesinos-Lopez et al., 2019).

The best prediction quality in terms of accuracy and stability of results was obtained with SVM and GB for subset sizes equal or larger than 500 SNPs. These two non-parametric methods outperformed GBLUP and regularized methods (RR, LR, and ENET). This could be due to the ability of non-parametric models to capture interactions among predictor variables and non-linear relationships with the target variable without explicitly modeling these interactions or functional forms (Gianola et al., 2006; Gianola and van Kaam, 2008). They are potentially able to capture complex signals from the data and deliver a better predictive accuracy, even if the trait is under additive gene action (Perez-Rodriguez et al., 2012, 2013). With large subset sizes (i.e., 1,000–1,500 SNPs), the filter method had no appreciable influence on prediction quality, which was almost the same as with random SNP selection. However, FS was essential for the implementation of both methods, which had numerical problems when all 9,523 SNPs were included in the analysis. With small subset sizes (i.e., equal or smaller than 250 SNPs), the FS algorithm used had a huge impact. In fact, prediction quality was poor when tree-based methods or random selection were used for FS with any learner, but it was comparable to the one attained with larger SNP subsets when spearcor or mrmr were implemented for SNP pre-selection combined or not with embedded methods. Those filter methods also led to more stable results (i.e., smaller IQR of SC).

Regularized methods (ENET, LR, and RR) with or without embedded FS followed the same pattern with respect to subset size (Figure 3). When no SNP pre-selection was performed or subset size was equal or larger than 750 SNPs, prediction accuracy was not different from the one attained with GBLUP, although results were more stable with the latter (Figure 2). In this situation (i.e., with medium-large subset sizes), FS would reduce computation time and resources, but would not improve over the predictions from regularized regression. Like for SVM and GB, when subset size was smaller than 500 SNPs, the filter method had a marked influence on prediction quality, with spearcor and mrmr being the only methods that produced a prediction quality comparable to the one obtained when all 9,523 SNPs were used.

Results regarding the stability of FS (Tables 1–4) were quite clear. Stability generally increased with the subset size. Stability of tree-based methods for FS (univ.dtree and cforest) was considered null. The FS methods that produced the best prediction quality (spearcor and mrmr) even with small subset sizes (i.e., 50 or 250 SNPs) were also the ones that showed stable compositions of SNP subsets, insensitive to changes in datasets. Univariate FS was computationally fast, but it does not account for the potential correlation between features. This could lead to similar ranking scores of correlated features that would potentially be selected together leading to increased redundancy in the feature subset retained. The similar performance of spearcor and mrmr methods, one univariate and the other multivariate, could be due to the fact that most correlated and less variable SNPs were excluded in the previous step of SNP quality control, before the FS process. This could have masked the potential differences in stability resulting from accounting for correlations between features or not. However, when combined with regularized methods (ENET and LR), the SNP subsets obtained with spearcor were more trimmed by the regularization than those obtained with mrmr, suggesting that some redundancies remained. With this two-step approach, spearcor seems preferable over mrmr, because of its much lower requirements in computation time and resources.

Elastic net and LR had the same stability values within subset size, despite different underlying SNP selection strategies. According to Nogueira et al. (2018), when features are correlated, LR would tend to select different features in different subsets. Elastic net might increase stability. However, as stated above, we used 9,523 out of 45,610 SNPs, with a PC smaller than 0.8 in order to reduce computation requirements of the ML algorithms. This pre-selection may have reduced redundancy, which produces instability (Gulgezen et al., 2009), reducing the differences between both regressors.

In conclusion, both accuracy and stability of results and of feature selector should be accounted for when constructing a model for prediction. In the case of prediction of RFI in growing pigs, different FS algorithms performing similarly well for prediction had wide differences in terms of stability. This issue can have important consequences on the interpretability and reproducibility of results and should be considered as an additional criterion to consider when evaluating FS methods. Elastic net, LR, and RR did not outperform GBLUP when the 9,523 SNPs were used for prediction or when they were pre-selected according to some criteria. With these learners, when the subset size was small (50–250 SNPs), only SNPs pre-selected with mrmr or spearcor produced prediction accuracies comparable to that of GBLUP with all the SNPs, and good FS stabilities. Thus, a strong SNP pre-selection could be performed to reduce computation requirements for regularized regression. Nevertheless, the best prediction quality in terms of accuracy and stability was obtained with the ML approaches (SVM and GB) using 500 or more SNPs pre-selected with mrmr or spearcor as predictor variables. Thus, the use of low-density SNP chips for GS seems feasible. Finally, when SNP quality control includes removing highly correlated SNPs, SC is recommended for FS over mrmr because of its simplicity and small requirements in computation time and resources.

Results and conclusions for ADG were consistent with the ones obtained for RFI. They are provided in Supplementary Material.

## Data Availability Statement

The data analyzed in this study is subject to the following licenses/restrictions: The datasets presented in this article are not readily available because of containing information that could be used by commercial competitors. Topigs Norsvin (Beuningen, Netherlands) fully agrees with transparency in science and welcomes alternative analyses of the data with a gatekeeper, a trusted person, who considers the relevance and the motives of the people interested in the data. Requests to access these datasets should be directed to Dr. Rob Bergsma (Rob.Bergsma@topigsnorsvin.com).

## Author Contributions

MP and LT conceived and designed the study, carried out the analyses, and wrote the original draft. RB prepared and provided the raw data. DG and HG provided critical insights and gave methodological suggestions. All authors discussed the results, reviewed, and approved the final manuscript.

### Conflict of Interest

RB is a member of Topigs Norsvin’s (Beuningen, Netherlands) staff.The remaining authors declare that the research was conducted in the absence of any commercial or financial relationships that could be construed as a potential conflict of interest.

## Supplementary Material

Supplementary Table 1 Stability of feature selection methods for prediction of average daily gain with Elastic Net.

Supplementary Table 2 Stability of feature selection methods for prediction of average daily gain with LASSO regression.

Supplementary Table 3 Stability of filter methods for prediction of average daily gain.

Supplementary Table 4 Mean of the Pearson and Spearman correlation over all pairs of feature scores in the 10-outer training sets obtained with different filter methods for feature selection for prediction of average daily gain.

Supplementary Figure 1 Boxplots for the Spearman correlation for average daily gain obtained in 10-outer testing sets with a Genomic Best linear Unbiased Predictor (GBLUP), Elastic Net (ENET), Least Absolute Shrinkage and Selection Operator Regression (LR), and Ridge Regression (RR) with no feature selection method used to select the SNPs.

Supplementary Figure 2 Boxplots for the Spearman correlation for average daily gain obtained in 10-outer testing sets with Support Vector Machine for regression (SVM), Gradient Boosting (GB), Elastic Net (ENET), Least Absolute Shrinkage and Selection Operator Regression (LR), and Ridge Regression (RR) with 50, 250, 500, 750, 1,000 and 1,500 SNP subsets selected with different filter methods. Filter methods: Maximum relevance minimum redundancy (mrmr), Random forest (cforest), Spearman’s correlation (spearcor), Univariate decision tree (univ.dtree) and random selection (random)

Supplementary Figure 3 Left and middle panels: Boxplots for Nogueira et al. (2018) stability estimator obtained for average daily gain from embedded methods implemented with different SNP subsets sizes and several filter methods in 10 outer-training folds. Right panel: Boxplots for Nogueira et al. (2018) stability estimator of different SNP subset sizes obtained with different filter methods in the 10-outer training folds. Subsets sizes: 50, 250, 500, 750, 1,000 and 1,500 SNPs. Filter methods: Maximum relevance minimum redundancy (mrmr), Random forest (cforest), Spearman’s correlation (spearcor), Univariate decision tree (univ.dtree) and random selection (random).
