# Supplementary material for: Feature Selection Stability and Accuracy of Prediction Models for Genomic Prediction of Residual Feed Intake in Pigs Using Machine Learning
Source: Front Genet. 2021 Feb 22;12:611506. doi: 10.3389/fgene.2021.611506 (PMC7938892; doi:10.3389/fgene.2021.611506)
Supplement: Supplementary Table 1 — Stability of feature selection methods for prediction of average daily gain with Elastic Net. [file Data_Sheet_1.pdf]

SUPPLEMENTARY MATERIAL

Supplementary Table 1. Stability of feature selection methods for prediction of average daily gain with Elastic Net

Subset size[1]	Filter[2]	NSNPs[3]	PDF[4]	medianSel[5]	IQRSel[6]	NOG[7]
50	mrmr	500	0.33	50	0	0.46
	cforest	464	0.88	46	5	0.03
	spearcor	429	0.27	43	2	0.55
	univ.dtree	247	0.98	25	14	0.00
	random	188	0.99	21	3	0.00
250	mrmr	1953	0.29	196	3	0.51
	cforest	1633	0.74	178	57	0.06
	spearcor	1635	0.28	164	7	0.53
	univ.dtree	901	0.90	93	10	0.01
	random	876	0.92	92	9	0.01
500	mrmr	3339	0.27	336	11	0.54
	cforest	2037	0.65	206	8	0.10
	spearcor	2685	0.28	279	18	0.53
	univ.dtree	1546	0.84	163	18	0.03
	random	1399	0.86	156	45	0.02
750	mrmr	3588	0.27	344	15	0.56
	cforest	2493	0.61	256	19	0.12
	spearcor	2883	0.27	281	22	0.56
	univ.dtree	1849	0.78	193	42	0.04
	random	1872	0.81	202	47	0.03
1000	mrmr	3865	0.25	386	11	0.58
	cforest	2902	0.58	288	19	0.13
	spearcor	3269	0.25	332	24	0.59
	univ.dtree	2106	0.74	196	55	0.05
	random	2334	0.76	251	16	0.04
1500	mrmr	4559	0.25	454	13	0.59
	cforest	3480	0.53	354	30	0.16
	spearcor	3986	0.25	393	27	0.59
	univ.dtree	2406	0.68	246	71	0.07
	random	2545	0.69	297	89	0.06
9523	none	3764	0.25	363	16	0.59

[1]Subset size = number of selected features

[2]Filter method = Maximum relevance minimum redundancy (mrmr); Random forest (cforest); Spearman's correlation (spearcor); Univariate decision tree (univ.dtree); Random selection (random)

[3]NSNPs = Total number of SNPs pre-selected in the 10 subsets

[4]PDF = Proportion of distinct features in the 10 subsets

[5]medianSel =Mean number of selected SNPS in the 10 subsets

[6]IQRSel = Interquartile range of the number of selected SNPS in the 10 subsets

[7]NOG = Nogueria et al. (2018) stability estimator

Supplementary Table 2. Stability of feature selection methods for prediction of average daily gain with LASSO regression

Subset size[1]	Filter[2]	NSNPs[3]	PDF[4]	medianSel[5]	IQRSel[6]	NOG[7]
50	mrmr	500	0.33	50	0	0.46
	cforest	473	0.88	48	2	0.03
	spearcor	433	0.27	43	3	0.55
	univ.dtree	281	0.99	33	7	0.00
	random	209	1.00	26	5	0.00
250	mrmr	1922	0.29	194	6	0.51
	cforest	1664	0.74	169	20	0.06
	spearcor	1576	0.28	159	3	0.53
	univ.dtree	985	0.91	102	30	0.01
	random	1066	0.92	119	18	0.01
500	mrmr	2911	0.27	292	5	0.55
	cforest	2451	0.68	249	16	0.08
	spearcor	2464	0.28	244	15	0.54
	univ.dtree	1683	0.84	175	70	0.03
	random	1583	0.86	147	37	0.02
750	mrmr	3720	0.26	377	28	0.57
	cforest	2838	0.64	300	54	0.10
	spearcor	3064	0.27	308	20	0.55
	univ.dtree	1963	0.79	193	42	0.04
	random	2103	0.81	227	53	0.03
1000	mrmr	4241	0.26	435	41	0.56
	cforest	3223	0.61	322	54	0.12
	spearcor	3489	0.27	350	22	0.56
	univ.dtree	2156	0.74	223	23	0.05
	random	2195	0.76	227	18	0.04
1500	mrmr	5207	0.25	508	49	0.57
	cforest	3577	0.54	373	59	0.15
	spearcor	4375	0.26	439	20	0.56
	univ.dtree	2676	0.68	284	21	0.07
	random	2807	0.69	287	73	0.06
9523	none	4696	0.25	461	30	0.57

[1]Subset size = number of selected features

[2]Filter method = Maximum relevance minimum redundancy (mrmr); Random forest (cforest); Spearman's correlation (spearcor); Univariate decision tree (univ.dtree); Random selection (random)

[3]NSNPs = Total number of SNPs pre-selected in the 10 subsets

[4]PDF = Proportion of distinct features in the 10 subsets

[5]medianSel =Mean number of selected SNPS in the 10 subsets

[6]IQRSel = Interquartile range of the number of selected SNPS in the 10 subsets

[7]NOG = Nogueria et al. (2018) stability estimator

Supplementary Table 3. Stability of filter methods for prediction of average daily gain

Subset size[1]	Filter method[2]	NSNPs[3]	PDF[4]	NOG[5]
50	mrmr	500	0.33	0.46
	cforest	500	0.88	0.03
	spearcor	500	0.25	0.61
	univ.dtree	500	0.97	0.00
	random	500	0.98	0.00
250	mrmr	2500	0.25	0.58
	cforest	2500	0.75	0.05
	spearcor	2500	0.21	0.67
	univ.dtree	2500	0.87	0.00
	random	2500	0.88	0.00
500	mrmr	5000	0.22	0.65
	cforest	5000	0.69	0.05
	spearcor	5000	0.19	0.70
	univ.dtree	5000	0.77	0.01
	random	5000	0.79	0.00
750	mrmr	7500	0.20	0.68
	cforest	7500	0.63	0.05
	spearcor	7500	0.18	0.73
	univ.dtree	7500	0.70	0.01
	random	7500	0.71	0.00
1000	mrmr	10000	0.19	0.70
	cforest	10000	0.57	0.05
	spearcor	10000	0.18	0.74
	univ.dtree	10000	0.62	0.01
	random	10000	0.64	0.00
1500	mrmr	15000	0.18	0.72
	cforest	15000	0.48	0.05
	spearcor	15000	0.17	0.74
	univ.dtree	15000	0.51	0.02
	random	15000	0.52	0.00

[1]Subset size = number of selected features

[2]Filter method = Maximum relevance minimum redundancy (mrmr); Random forest (cforest); Spearman's correlation (spearcor); Univariate decision tree (univ.dtree); Random selection (random)

[3]NSNPs = Total number of SNPs selected in the 10 subsets

[4]PDF = Proportion of distinct features in the 10 subsets

[5]NOG = Nogueria et al. (2018) stability estimator

Supplementary Table 4. Mean of the Pearson and Spearman correlation over all pairs of feature scores in the 10-outer training sets obtained with different filter methods for feature selection for prediction of average daily gain

Filter method[1]	Pearson	Spearman
mrmr	0.888	0.907
cforest	0.039	0.024
spearcor	0.897	0.852
univ.dtree	0.032	0.030
random	0.001	0.001

[1]Filter method = Maximum relevance minimum redundancy (mrmr); Random forest (cforest); Spearman's correlation (spearcor); Univariate decision tree (univ.dtree); Random selection (random)

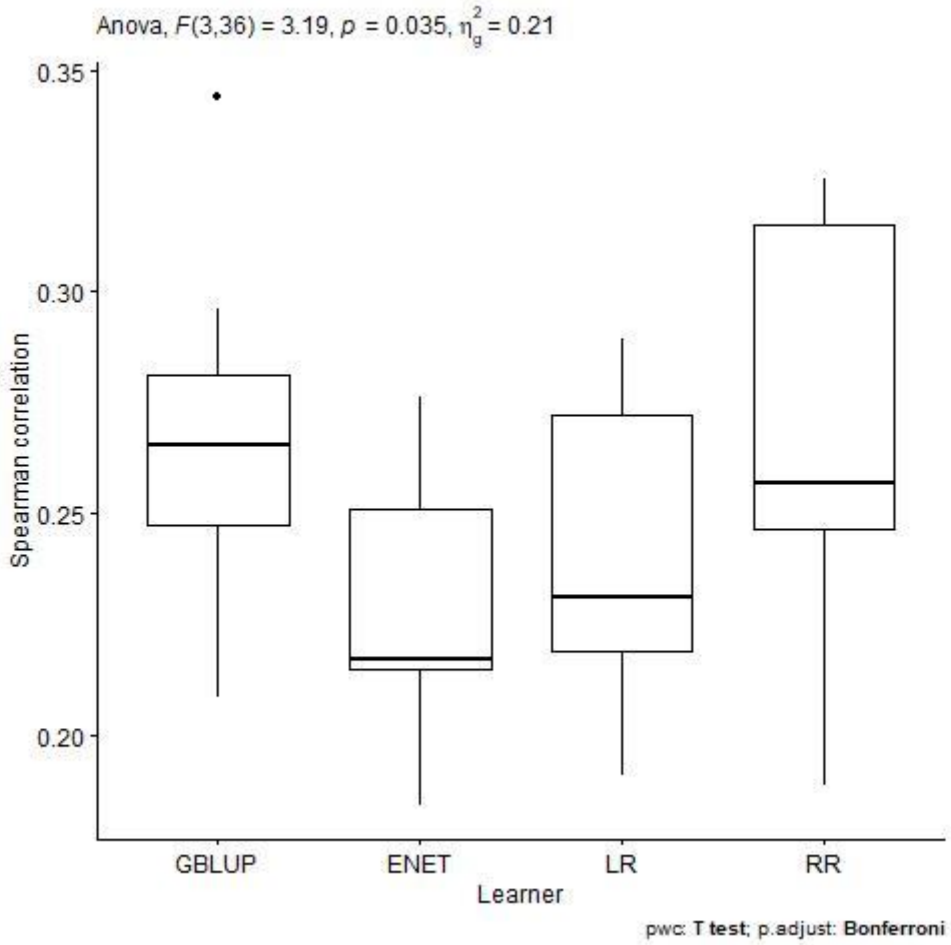

Supplementary Figure 1. Boxplots for the Spearman correlation for average daily gain obtained in 10-outer testing sets with a Genomic Best linear Unbiased Predictor (GBLUP), Elastic Net (ENET), Least Absolute Shrinkage and Selection Operator Regression (LR), and Ridge Regression (RR) with no feature selection method used to select the SNPs

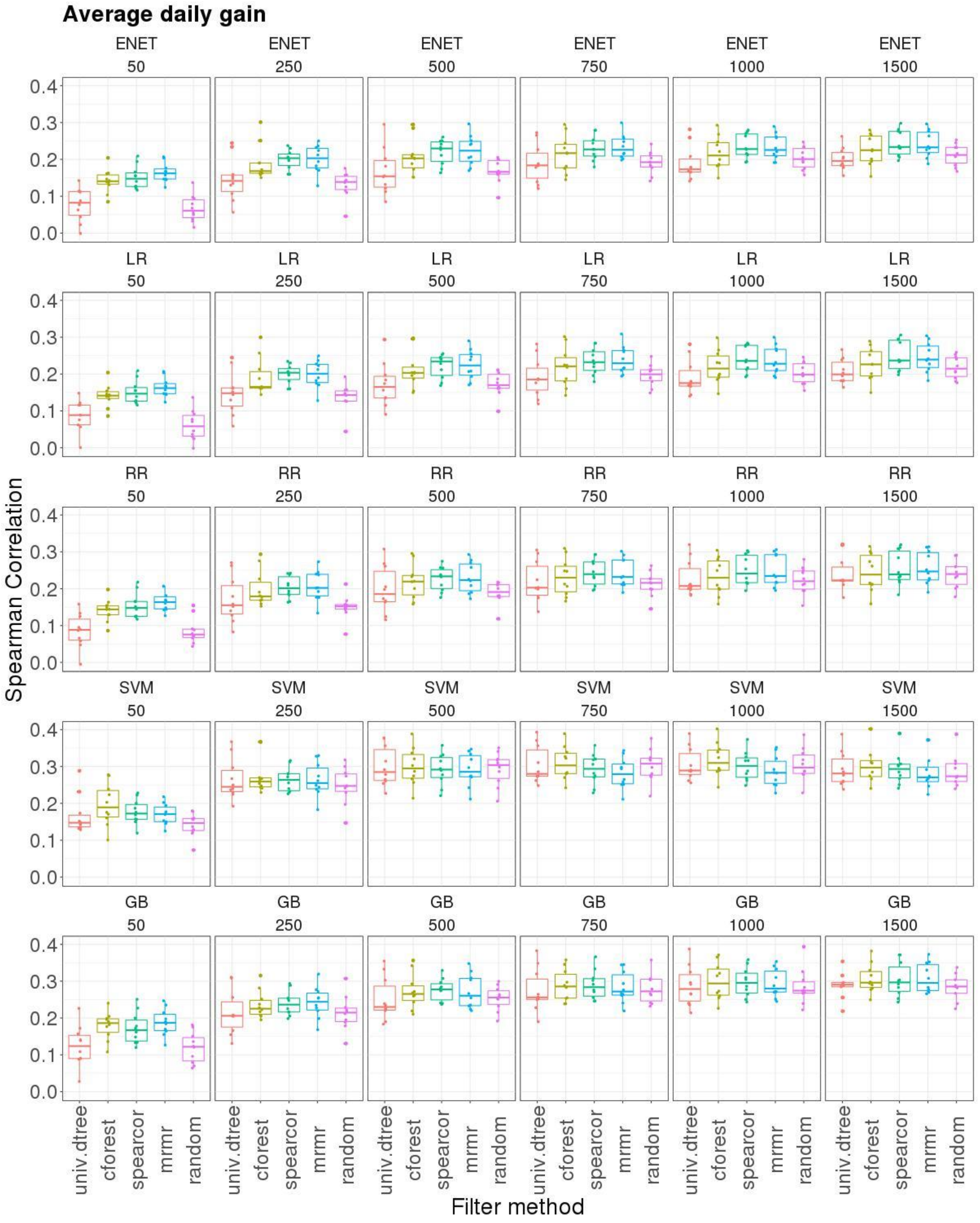

Supplementary Figure 2. Boxplots for the Spearman correlation for average daily gain obtained in 10-outer testing sets with Support Vector Machine for regression (SVM), Gradient Boosting (GB), Elastic Net (ENET), Least Absolute Shrinkage and Selection Operator Regression (LR), and Ridge Regression (RR) with 50, 250, 500, 750, 1,000 and 1,500 SNP subsets selected with different filter methods. Filter methods: Maximum relevance minimum redundancy (mrmr), Random forest (cforest), Spearman's correlation (spearcor), Univariate decision tree (univ.dtree) and random selection (random)

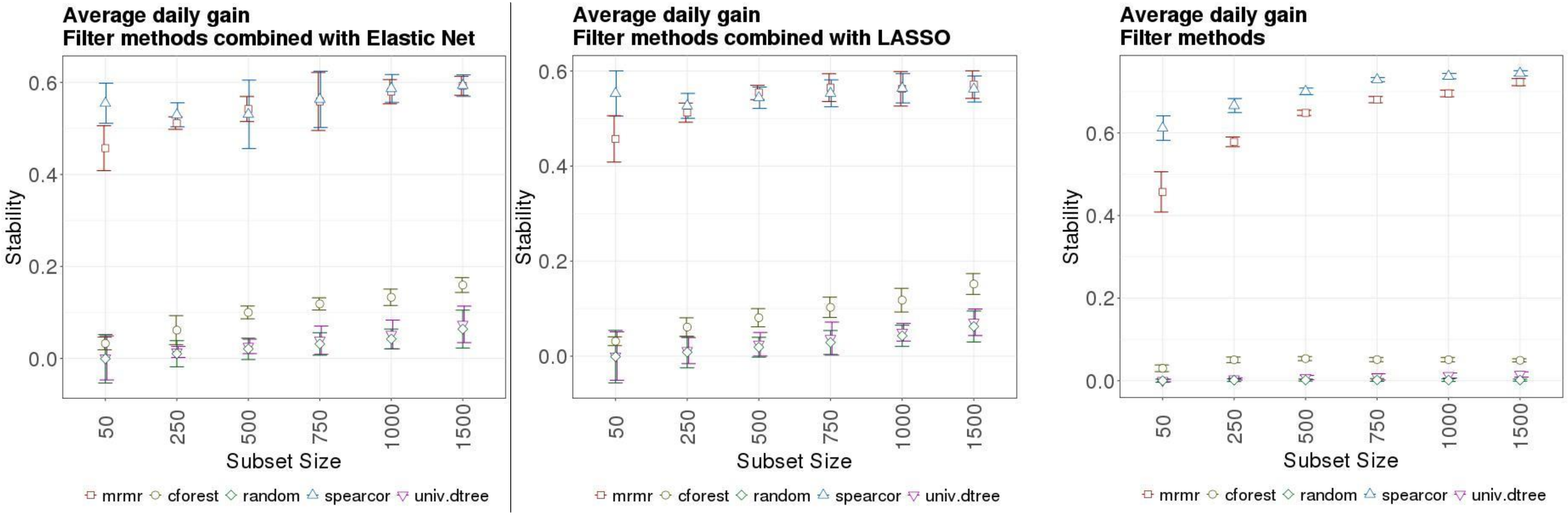

Supplementary Figure 3. Left and middle panels: Boxplots for Nogueira et al. (2018) stability estimator obtained for average daily gain from embedded methods implemented with different SNP subsets sizes and several filter methods in 10 outer-training folds. Right panel: Boxplots for Nogueira et al. (2018) stability estimator of different SNP subset sizes obtained with different filter methods in the 10-outer training folds. Subsets sizes: 50, 250, 500, 750, 1,000 and 1,500 SNPs. Filter methods: Maximum relevance minimum redundancy (mrmr), Random forest (cforest), Spearman's correlation (spearcor), Univariate decision tree (univ.dtree) and random selection (random)
